# The BEHAVE application as a tool to monitor inclusive interventions for subjects with neurodevelopmental disorders

**DOI:** 10.3389/fpsyg.2022.943370

**Published:** 2023-01-18

**Authors:** Gianluca Merlo, Antonella Chifari, Giuseppe Chiazzese, Paola Denaro, Noemi Firrera, Nicola Lo Savio, Simona Patti, Luisa Palmegiano, Davide Taibi, Luciano Seta

**Affiliations:** ^1^Istituto per le Tecnologie Didattiche, Consiglio Nazionale delle Ricerche, Palermo, Italy; ^2^Istituto Tolman, Palermo, Italy

**Keywords:** neurodevelopmental disorders, applied behavior analysis, communication impairments, technologies for monitoring, inclusive intervention

## Abstract

In the last few years, many educational and therapeutic interventions for young people with neurodevelopmental disorders are based on systematic monitoring of the outcomes. These interventions are typically conducted using single-case experimental designs, (SCEDs) a set of methods aimed at testing the effect of an intervention on a single subject or a small number of subjects. In SCEDs, an effective process of decision-making needs accurate, precise, and reliable data but also that caregivers and health professionals can gather information with minimal effort. The use of Information Communication Technologies in SCEDs can support the process of data collection and analysis, facilitating the collection of accurate and reliable data, providing reports accessible also by non-experts, and promoting interactions and sharing among clinicians, educators, and caregivers. The present paper introduces the BEHAVE application, a web-based highly customizable application, designed to implement SCEDs, supporting both data collection and automatic analysis of the datasets. Moreover, the paper will describe two case studies of kindergarten children with neurodevelopmental disorders, highlighting how the BEHAVE application supported the entire process, from data collection in multiple contexts to decision-making based on the analysis provided by the system. In particular, the paper describes the case studies of Carlo and Dario, two children with severe language and communication impairments, and the inclusive education interventions carried out to maximize their participation in a typical home and school setting increasing their mand repertoire. Results revealed an increase in the mand repertoire in both children who become able to generalize the outcomes to multiple life contexts. The active participation of the caregivers played a crucial role in the ability of children to use the learned skills in settings different from the ones they were learned in.

## 1. Introduction

Single-case experimental design (SCED) is an expression used to indicate a class of research designs, characterized by repeated observations of a single entity (generally an individual, but can be also a group, a classroom, a school, or a hospital) in a fixed period during at least a variable is manipulated, generally the treatment.

SCEDs are often contrasted with randomized control trials (RCTs), in which two or more groups, control vs. experimental groups, are compared with the aim to establish the effects of an intervention using standardized and validated instruments. For many years, RCTs were the “gold standard” for experimental studies. Recently, a growing number of works have criticized the general usefulness of these clinical trials to discern the impact on the individual health of some specific treatments (Jacobson and Christensen, 1996; Westen and Bradley, 2005; Wachtel, 2010; Branch, 2014; Perone, 2019). An RCT study can capture the “average” effect but is not suitable to determine the causal/functional relationship between a treatment and observed change for one individual: “managers and trialists may be happy for treatments to work on average; patient’s doctors expect to do better than that” (Evans, 1995; cited by Vlaeyen et al., 2020, p. 659). SCEDs are especially suitable for establishing evidence of intervention efficacy and conducting pilot investigations, also for large-scale causal studies (Smith, 2012; Natesan Batley et al., 2020). Although the methodology underpinning SCED has a long history, at least since the work of experimental psychology based on the behavioral approach and operant conditioning (Skinner, 1938; Sidman, 1952, 1960; Skinner, 1956; Shapiro, 1966) recent epistemological and methodological developments have brought this type of study back into the mainstream.

The use of SCEDs is historically linked to the study of human and animal behavior and the search for causal or functional relationships between the manipulation of the subject’s living environment and observable changes in his/her behaviors (Tate and Perdices, 2019; Vlaeyen et al., 2020; Kazdin, 2021). This methodology is therefore frequently used when it comes to testing the effectiveness of therapeutic interventions in developmental disorders. For example, in a recent systematic review of behavior analytic interventions for young children with intellectual disabilities (Ho et al., 2021) of the 49 studies included, only three (6%) were group-design studies, and the rest used single-case design methodology. Moreover, SCED appears as the prevalent methodology (Cannella-Malone et al., 2021) to monitor problem behaviors related to the most common neurodevelopmental disorders (Horner et al., 2005; Odom et al., 2005; Cook and Cook, 2013; Cook and Odom, 2013).

For this type of issue, the interventions are often tailored to the specific characteristics of the individual and based on the assumption that behavioral change can be the result of a learning process. The comparison between groups can be affected by important biases, related to the difficulty to have homogeneous groups, the intervention of spurious variables, the interpretation of the results in terms of efficacy on a single individual, and the translation of correlation in a functional relationship.

SCEDs can also be affected by the risk of bias. The most frequent risks are related to the inability to conceal certain elements of the research design from study participants, researchers, and individuals collecting outcome data. Another frequent risk of bias is related to the lack of clear documentation of fidelity to the experimental procedures (Smith et al., 2022).

The use of ICTs in SCEDs is relevant to reduce the risk of bias supporting the process of accurate and reliable data collection (Spachos et al., 2014). Moreover, ICTs could promote the generation of reports accessible also by non-experts, and the interaction and sharing among the different caregivers.

The present paper introduces the BEHAVE application as a tool to promote the culture of evidence-based principles both in clinical and educational contexts, facilitating the process of monitoring and management of the problem behavior linked to neurodevelopmental disorders (NDDs) and providing users with an easy way to gather data and evaluate the effect size of the behavioral interventions.

In particular, the paper will introduce the BEHAVE web application as a technological tool supporting an ABA inclusive intervention applied to two kindergarten children with autism and severe language and communication impairments. First, theoretical points of departure about autism spectrum disorder (ASD) and language and communication impairments will be described. Then the paper will describe the two mentioned case studies, highlighting how the BEHAVE application supported the entire process, from data collection in multiple contexts to decision-making based on the analysis provided by the system.

## 2. Autism spectrum disorder and language and communication impairments

According to DSM-5 (American Psychiatric Association, 2013), the diagnostic class of neurodevelopmental disorders (NDDs) comprises disorders that arise during the developmental period characterized by personal, social, scholastic, or occupational difficulties. The phenotypes of NDDs are very heterogeneous including for example intellectual disabilities, autism spectrum disorders (ASD), attention-deficit/hyperactivity disorders, communication disorders, neurodevelopmental motor disorders, and specific learning disorders. The etiology of these disorders is considered multifactorial (Guo et al., 2018) involving, among others, genetic, perinatal, endocrine, and psychosocial risk factors. The prevalence of NDDs is highly variable changing as a function of the disorder typology, socioeconomic factors, and sex. For example, intellectual disabilities range from 0.3 to 8% according to the severity (Simonoff, 2015), speech disorders from 2 to 31% (Norbury and Paul, 2015), and autism spectrum disorders from 0.6 to 1% in developed countries (Williams et al., 2006; Matson and Kozlowski, 2011) to 3% in South Korea and Japan (Elsabbagh et al., 2012).

One of the most studied NDDs is ASD. According to a behavioral perspective, ASD is a syndrome characterized by behavioral deficits and excesses, which have a neurological basis, but can be modified as a result of specific interactions with the environment (Martin and Pear, 2019). ASDs are biologically determined neurodevelopmental conditions that generally begin in the first 3 years of life and accompany the individual throughout the life cycle.

The predominantly affected areas are those related to communication and social interaction and the presence of restricted and repetitive patterns of behavior, interests, or activities (American Psychiatric Association, 2013). Deficits in these areas can be expressed in very different ways from one person to another, can vary over time depending on the interaction with the context, and can foster the emergence of different types of emotional, social, and behavioral disorders. Deficiencies in communication skills are some of the most common deficits in people with autism spectrum disorders (Peeters and Gillberg, 1999). Communication skills are fundamental for good children’s social interaction within their living environment. Deficiencies in these skills can lead to the emergence of problem behaviors, which sometimes represent substitute modes of communication (Cooper et al., 2007; Moderato and Copelli, 2010; Sundberg and Sundberg, 2011).

Several studies have stressed the importance of early identification of subclinical signs of autism to facilitate access to early treatment and improve prognosis (Daniels et al., 2014; Costanzo et al., 2015). Indeed, untimely interventions put children at risk of developing sleeping disorders (Kamara and Beauchaine, 2020), being physically and sexually abused (Balogh et al., 2001; Reiter et al., 2007; Jawaid et al., 2012), committing suicide (Lunsky, 2004) or violent crimes (Lundström et al., 2014).

Behavioral interventions based on applied behaviour analysis (ABA) now represent some of the best treatments available for developing communication skills in people with ASD (Peters-Scheffer et al., 2011; Reichow et al., 2018; Makrygianni et al., 2018; Yu et al., 2020).

ABA is a science based on learning principles that aim to predict and influence behavior to promote socially meaningful behavior (Cooper et al., 2007). ABA enables the implementation of individualized interventions to develop deficient skills and reduce barriers to learning in individuals with autism spectrum disorders through the identification and modification of contextual variables that influence behavior and the application of systematically applied procedures.

Starting from the seminal work of Skinner (1957), behavior analysis has developed a functionalist approach to language analysis and verbal relations (Presti et al., 2002), which has allowed the definition of procedures that are effective in promoting communication skills in people with cognitive delays and disabilities (Presti et al., 2002; Carbone et al., 2010). Skinner (1957) considers language as learned behavior and defines verbal communication as an operant behavior of a speaker reinforced through the mediation of a listener, who has learned in the verbal community to provide appropriate consequences to the speaker’s behavior. From this perspective, the main focus is on the function as well as the form of the verbal relationship.

A taxonomy of responses called verbal operants is defined starting from the identified behavioral function. This system of analysis and classification has important implications (Sundberg and Michael, 2001) for the development of language and communication in individuals with autism spectrum disorder, both through the teaching of vocal language and through the use of alternative augmentative communication (AAC) systems, such as the use of gestures and equipment for the partial or total, temporary or permanent, compensation of severe difficulties in the emission of vocal language (Cafiero, 2009). The acquisition of verbal behavior promotes the development of cognitive, scholastic, and social skills (Sundberg and Michael, 2001).

Among verbal operants, mands are fundamental for the development of language and social interactions. Skinner (1957) defines the mand as “a verbal operant in which the response is reinforced by a characteristic consequence and is therefore under the functional control of relevant conditions of deprivation or aversive stimulation” (p. 35–36). Mand is a type of verbal behavior controlled by motivational operations. It allows the speaker to request what he needs and wants and is usually among the first forms of children’s communication (Sundberg, 2008). Hand signs (Carbone et al., 2010), pictures exchange communication system (Jurgens et al., 2009), and voice imitation training (Ross and Greer, 2003) are successful examples of strategies for vocal imitation mand teaching.

## 3. Technology to assess, monitor, and treat NDDs

Technologies-based monitoring practices are conceived as an evidence-based data collection process able to capture dynamic changes in psychological, cognitive, and behavioral outcomes and to support customized individual interventions (Bentley et al., 2018) in clinical practices. The spread of smart devices (such as mobile and wearable devices) has opened up scenarios where it is possible to reach the subjects by assessing them through observations in their natural environment. In particular, the development of new digital health solutions and services has allowed therapists to assess, monitor, and treat the patient by offering a more suitable and feasible digital intelligent health service.

A recent systematic review by Valentine et al. (2020) identifies how smart devices like tablets, smartphones, and wearable devices may be combined with apps, gaming applications, and video modeling behavioral training activities to support the assessment, diagnosis, treatment, and monitoring processes. Examples of clinical services supported by technologies are virtual reality assessment/therapy, telehealth assistance, computer-based assessment/therapies, and monitoring across multiple NDDs, especially ASD and ADHD disorders. The review also highlights the positive technological impact on clinical effectiveness, economic cost–benefit, and the user feasibility and acceptability of technology (Valentine et al., 2020).

The introduction of behavioral tracking with smart devices (Shiffman et al., 2008; Shingleton et al., 2016; Wichers and Groot, 2016), and the use of wearable biosensors for physiological assessment (Schlier et al., 2019) underpins the collection of repeated systematic observation over time (Bentley et al., 2018) and the advancement in the quantitative techniques used for SCED has enhanced and facilitated the interpretation of outcomes, the communication of the results between different subjects, and comparing the results using common, quantitative approaches (Barnard-Brak et al., 2022).

In the last few years, one of the prevalent technologies for developing behavioral monitoring applications according to SCED has been touch screen-based smart devices, and this trend is likely to continue in the coming years (Saini and Roane, 2018).

The development of these applications has followed two different paths. First, there are numerous research projects that have created tools (often with open-source code releases) designed with the purpose of providing practitioners with software to support data-driven decisions. Some non-exhaustive examples are the WHAAM application for monitoring subjects with ADHD (Spachos et al., 2014), BDataPro that supports behavioral data collection and visual analysis of the same (Zheng et al., 2022), BASE for implementing behavioral interventions at school (Chiazzese et al., 2019), and the AHA application for monitoring and evaluating the attention skills of subjects with ADHD engaged in the use of an augmented reality-based solution to stimulate reading and writing skills (Tosto et al., 2021).

Second, many software houses developed paid applications that could support a specific niche of health professionals working with neurodevelopmental disorders and, more generally, with behavioral disorders. An overview of existing commercial systems and their peculiarities is provided by Merlo (Merlo et al., 2020).

What emerges from the analysis of these applications is that, in general, they are inflexible and not customizable since they are often anchored to a specific theoretical and methodological approach. Moreover, in most cases, the software do not provide sophisticated tools for automatic data analysis, leaving users with the task of visually analyzing the data or exporting the collected dataset for later autonomous analysis.

The BEHAVE system that will be presented below aims to address these limitations by providing its users with a highly customizable tool so that each practitioner can use it in accordance with his or her own approach. In addition, the system is capable of generating reports based on automatic statistical analyses that can be understood even by those without strong statistical skills.

The BEHAVE application is a tool that was created as the main output of a project funded by the European Commission in 2017 (2017-1-IT02-KA201-036540) inside a KA2 Strategic Partnership for school education Erasmus+. More in-depth, the project was aimed at enhancing the experience and expertise of health professionals, teachers, parents, and caretakers in the management of behavioral interventions, according to the idea that the SCED methodology is a good practice to measure processes and procedures and to support the implementation of evidence-based practices, clarifying what are the most effective strategies case by case.

As mentioned above, the most innovative feature of the BEHAVE application is the opportunity to support the management of selected problem behaviors through the creation of custom measures, the operational definition of behavior, the collection of behavioral data, and the comparison between phases (e.g., baseline and intervention) through both the visual comparison of data represented by scatter plots and statistical analyses that are generated automatically.

In particular, the application can identify the best algorithm of effect size among those developed by Parker et al. (2011) and Allison and Gorman (1993), returning the effect size of the intervention displayed simply and clearly, through a speedometer that guides to the meaning of the data collected even the least experienced user. Providing users with the data and their analysis in a simple and accessible way could thus facilitate the introduction of evidence-based scientific approaches in multiple contexts beyond the clinical one.

In the BEHAVE application, users can combine six different types of questions, called items, to collect data about behaviors. One of these is the “Direct observation item” useful to collect data on how long or how often a certain behavior occurs during the observation. For example, the frequency, duration, and intensity of specific behavior can be assessed when a student interrupts a class, leaves his seat, raises his hand, yells out an answer, or asks to go to the bathroom. Direct observation items support the frequency, duration, and interval recording. The choice of the recording procedure depends on the typology of behavior that caregivers want to observe.

Other measures are:

- The “Choice item” is useful when the question includes one or more answers among a group of predefined answers. A famous example of choice item with one answer allowed is the Likert scale, a scale composed of items to whom respondents must specify their level of agreement or disagreement on a symmetric agree-disagree scale.
- The “Number item” are questions that can be answered only with numbers. For example, these items can be useful to count how many times a child threw a pencil or other object at other pairs. A simple numeric item such as “How many times the child threw the pencil?” can be created to obtain a numerical answer.
- “Range items” are similar to number items but instead to accept any integer value, they accept only values included between a minimum and a maximum.
- “Four quadrant item” is used to measure at the same time two different dimensions. The measure is composed of two dimensions displayed on a cartesian plane. Users have to choose how to position themselves in the two dimensions.
- “Text items” are aimed at gathering qualitative data about a phenomenon through the generation of open-ended questions.

The BEHAVE application allows users to combine different already existing measures to create their favorite combination of items. Users can import and export the created measures and share them with others to support a community of practice.

Finally, the BEHAVE application makes available a set of education support tools to coach the users through video tutorials within a Moodle course, a user guide to introduce the BEHAVE functionalities, and contextual help during the navigation of the application. The BEHAVE application is free of charge and it is accessible at the URL: https://www.behaveproject.eu/.

## 4. Case studies

Carlo is a 3-year-old boy diagnosed with autism spectrum disorder who does not have vocal language. Dario is a 3 years and 5 months boy with a diagnosis of agenesis of the corpus callosum and autism spectrum disorder who presents unintelligible vocal language due to phonetic-phonological difficulties. The diagnoses were made at the territorial child neuropsychiatry services. The two children attend nursery school and have three weekly ABA therapy sessions of 1 h each. The initial assessment of the functional skills of the two children was carried out by cognitive behavioral psychotherapists through the Verbal Behavior Milestones Assessment and Placement Program (VB-MAPP, Sundberg, 2008). The assessment highlighted important deficiencies in verbal skills, play skills, and social skills in both children. As a consequence, the education intervention plans focused primarily on the increase of both the frequency and variety of unprompted mands emitted by the children.

The study was carried out following the ethical principles and codes of the institution that delivered the treatments which are based on national and international ethics codes (e.g., the code of ethics of Italian psychologists and the BACB’s Ethics Code for Behavior Analysts). Accordingly, approval by an ethics authority was not required. The informed consent form has been signed by the parents of the children involved in the study before the start of data collection.

### 4.1. Definition of the target behavior

**Figure 1:**
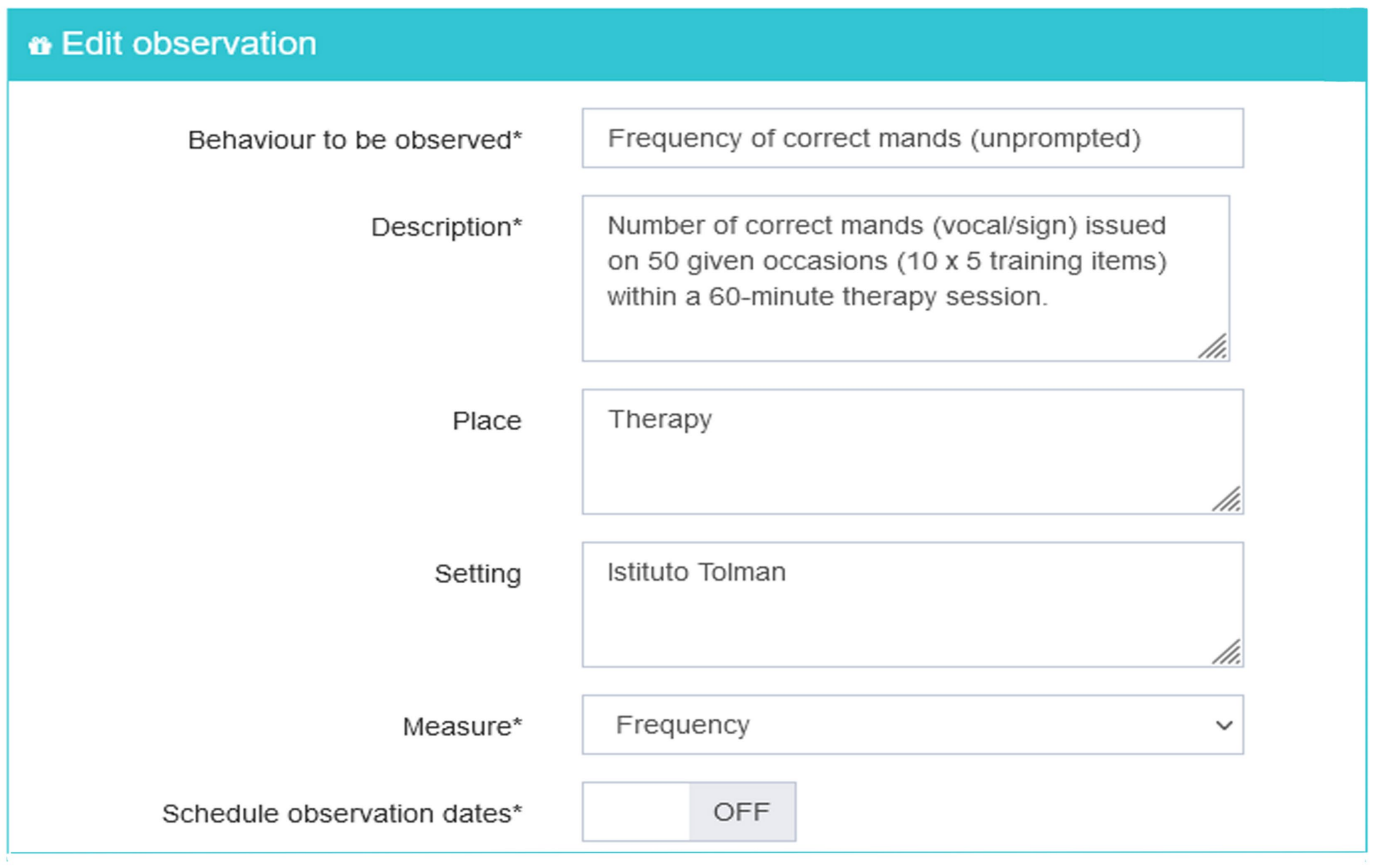
Screenshot of the target behaviors form of the BEHAVE application.

The dependent variable measured in this study was the number of mands made by Dario and Carlo for accessing edible and dynamic stimuli, as well as tangible reinforcing objects available in the environment. In the case of Dario, the presence of vocal language facilitated the development of vocal mand even if the vocalization was not used to make requests and was often not very intelligible. In the case of Carlo, alternative augmentative communication based on signs has been implemented to face the absence of vocal language. The definition of the target behavior is one of the first steps in the BEHAVE application. The therapist has to insert the behavior to be observed in the most accurate way specifying an operative description, the place, and the setting in which the behavior occurs. Figure 1 shows the definition of the target behaviors for the two case studies.

### 4.2. Measure creation and data recording

In the present study, data about the behaviors were collected through direct observations. In particular, a frequency direct observation measure was created with the BEHAVE application to measure how many times the number of mands occurred during the one-hour baseline and intervention sessions. The BEHAVE application was used to facilitate data collection in different contexts. In fact, in addition to the clinical context, the application facilitated the sharing of results between the figures involved in the therapeutic process, promoting the active participation of caregivers and maintaining high motivation for treatment. The BEHAVE application allowed the therapist to create a shareable URL to invite the children’s parents to collect data in the home context.

### 4.3. Design and procedure

The dependent variable measured in this study was the number of correct mands issued on 50 given occasions (10 × 5 training items) within a 60-min therapy session by Dario and Carlo.

An AB design was defined within the BEHAVE application and employed to evaluate the effectiveness of the intervention by comparing data gathered during the treatment and baseline phases. Data were collected through direct observations with the BEHAVE application in a different context by the therapists and the parents. During the therapy sessions, 35 observations were made, divided into 5 baseline and 30 training observations. The generalization phase included 10 observations during home sessions with parents. The minimum empirical value assumed by the dependent variable was 0 and the maximum value 50.

The intervention progress was periodically monitored through the scatter plots and automated analyses provided by the BEHAVE application.

#### 4.3.1. General procedure

**Figure 2:**
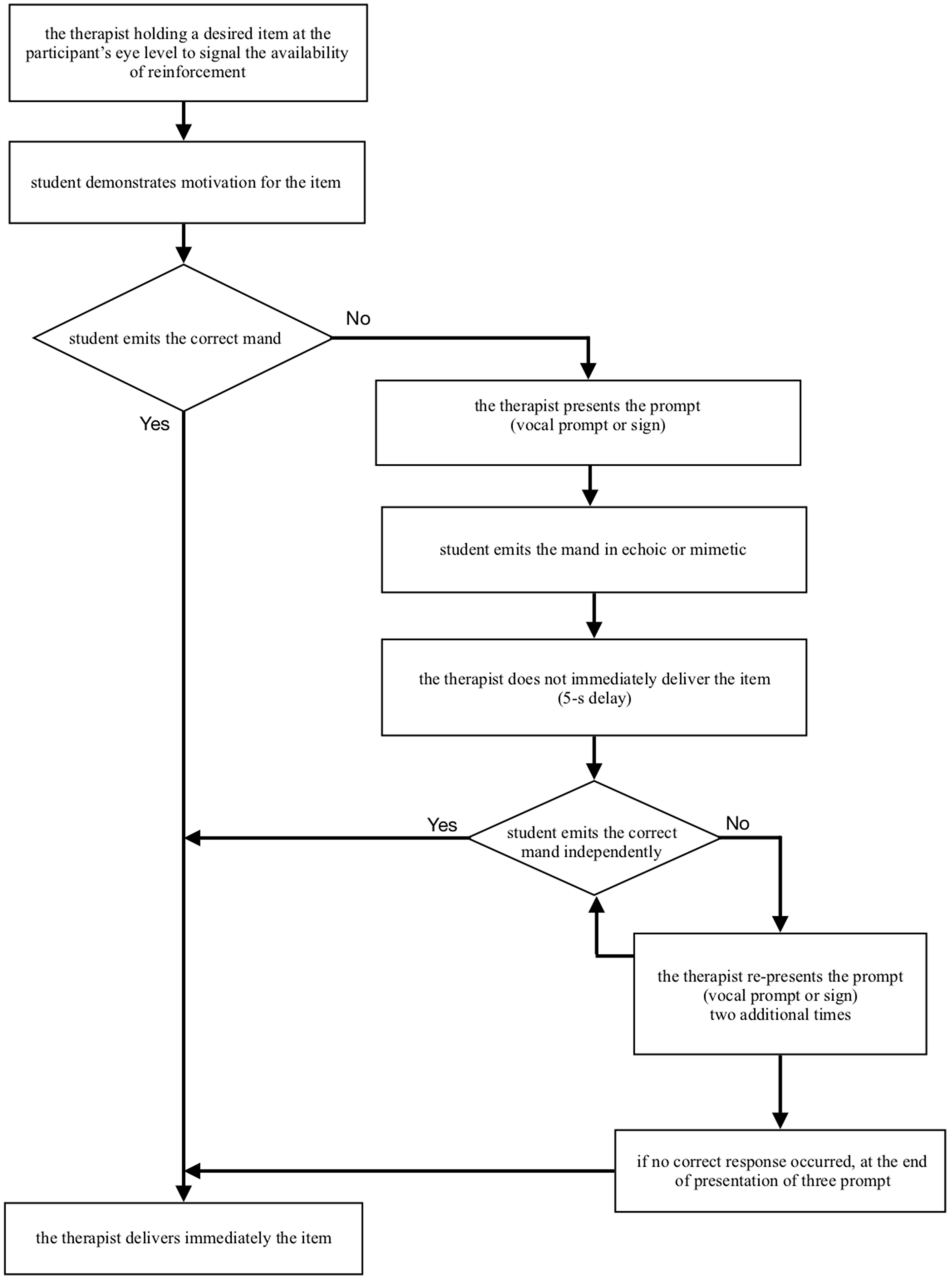
Flow chart describing the structure of a single therapy session with mand training.

According to Carbone et al. (2010), the first step of the teaching procedure is the selection of five items as target mand for each participant. This selection is the result of a previous assessment of the child’s motivation in which edible, dynamic stimuli and tangible objects (toys) are proposed to the child. The teaching sessions included 50 trials delivered in an individual setting: 10 opportunities (trials) were offered to request each motivating item, for a total of 50 trials per session. Targets were presented in a randomized rotation. At the beginning of each trial, the therapist presented the desired item to the child making him aware of the availability of the reinforcer. If the child did not show motivation toward the presented item within 5 s, the therapist presented the next target of the rotation. Otherwise, the therapist started a specific teaching procedure to maximize the child’s responses. Figure 2 details the structure of a single therapeutic session with mand training so as to facilitate the replicability of the study.

#### 4.3.2. Baseline

For the present study, the baseline data was gathered during 5 sessions in which each child had 50 opportunities to make mand (10 opportunities for each item) for their reinforcers within one therapy session. The mand emitted within 5 s of the presentation of the stimulus is considered a correct response. The response was considered incorrect in all other cases, including non-response. Both children at baseline scored zero.

#### 4.3.3. Mand training: Vocal prompt/physical prompt and delay prompt

In the present study, the mand training provided both children with a procedure that included the evocation of the motivation to request the desired object, the use of prompts to model the correct request, and the delivery of the stimulus corresponding to the motivation as a specific reinforcer. For Dario, the vocal prompt was used to model vocal communication, while for Carlo the physical prompt was used to model communication through signs. The training phase was initially carried out in the clinical setting by psychologists specialized in cognitive-behavioral psychotherapy and ABA. Moreover, the therapists implemented parent training to promote the generalization of learning in the natural environment.

When the child showed motivation for the items, the therapist provided the prompt (vocal for Dario and physical for Carlo’s sign manuals). If the child emitted the mand (in echoic for Dario and in mimetic for Carlo), the therapist waited 5 s (5-s delay). If during the 5-s delay the child performed the mand independently, the therapist delivered the desired item immediately and left it available for 30 s. Otherwise, the therapist repeated the procedure from the beginning. If the child did not make the correct response for three sequences of presentation of the procedure, the therapist delivered the desired item anyway (with a lower magnitude).

#### 4.3.4. The generalization of the mand skill with carers

When each child learned to emit the target mands for all of the 5 targets and reached 80% of correct mands in 3 consecutive therapy sessions (end of the intervention phase), parents were involved in 2 sessions of parent training and then they started to collect data through the BEHAVE application for 10 home sessions to monitor the generalization of the skill in the family’s natural context.

During parent training, parents were trained in the use of the behavior application and in manipulating the motivation of children to create opportunities for mand to be emitted. To do this, the parents were asked to create one-hour play sessions, in which the trained items were made available (on sight but not directly accessible), and the correct requests emitted by the children were recorded through BEHAVE application.

During periodic monitoring of data with the BEHAVE application, therapists noticed that while Dario immediately generalized the mand skill learned in the therapy session, Carlo was not able to generalize the skill independently. Carlo did not issue mand after three sessions of structured play at home and had therefore obtained a score of zero. For this reason, the therapists contacted Carlo’s parents to support them in teaching the mand to their child at home.

After the generalization phase at home, to evaluate the ecological impact of the training, the parents were asked to complete a short interview that investigated the following areas: (1) emission at the home of spontaneous mands for trained targets also when the motivating objects are not on sight in the environment; (2) emission in other contexts of spontaneous mands for trained targets; (3) contexts in which spontaneous mands for trained targets emerge; (4) increase in the communicative repertoire of communicative intentionality (presence of requests or approximations of requests for new items).

**Figure 3:**
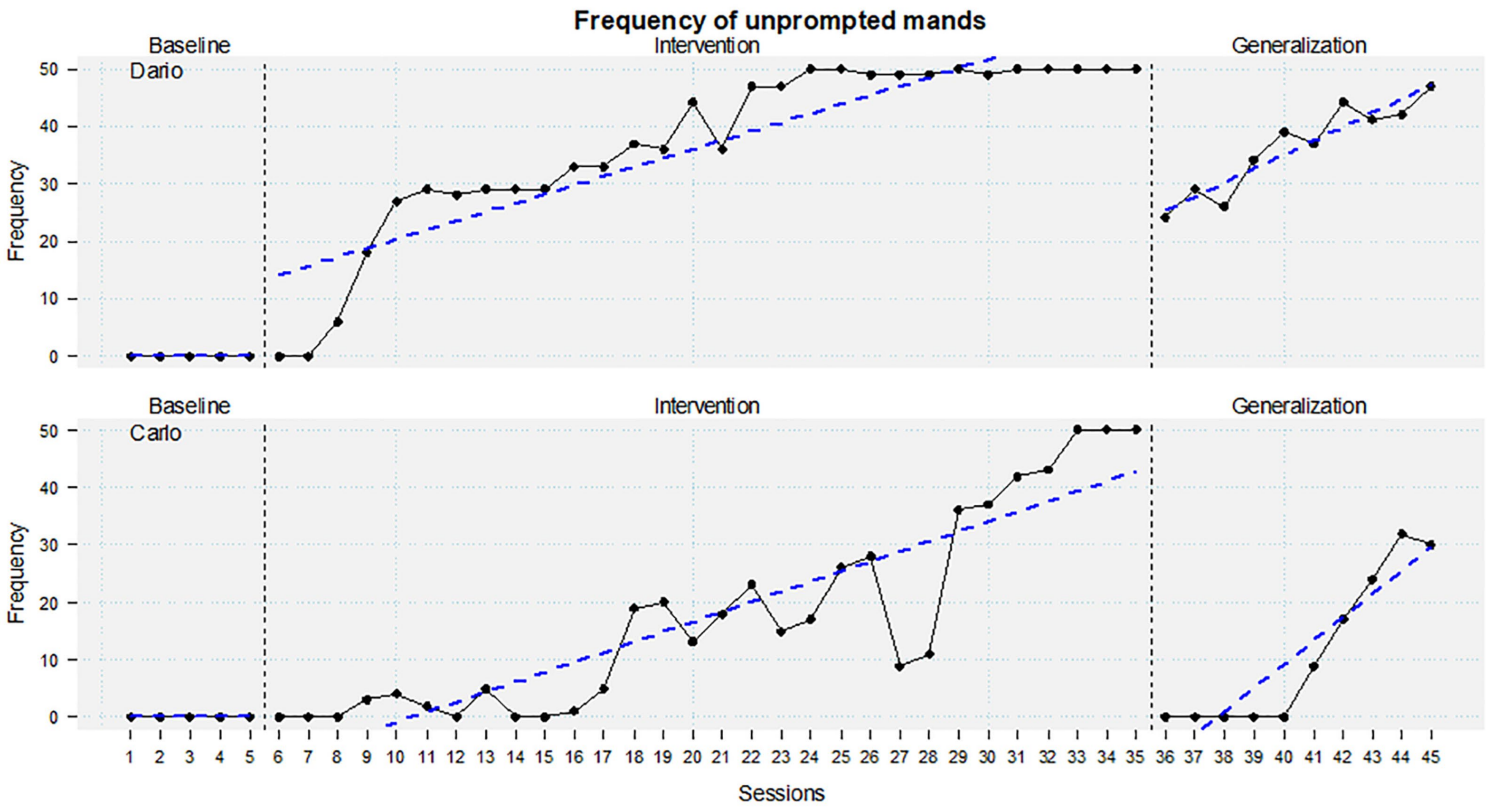
Scatterplot comparing baseline, intervention, and generalization of the unprompted mands for Carlo and Dario.

In conclusion, both children learned the mand in training and generalized them also at home with the caregivers, although at different times and modalities (Figure 3).

## 5. Results

The results showed an increase in the mand repertoire in both children and a generalization of this ability in various contexts.

Dario emits the first correct mands in the 8th therapy session and reaches 80% of the correct mands in the 20th session. Stability in the results is achieved by session 31. Carlo emits 80% of correct mands at the 32nd therapy session. The 100% of correct mands is achieved by session 33.

In addition, monitoring the data collected by caregivers through the BEHAVE application, the therapists observed that the results obtained in the generalization phase were different for the two children. Dario has immediately generalized the skills learned during the intervention, showing continuous growth in the frequency of mand issued at home. Carlo, on the other hand, generalized the skills acquired in the family context after a specific parent training.

The following paragraphs describe in detail the results obtained comparing baseline with intervention, and intervention with generalization.

### 5.1. Baseline versus intervention

A quasi-experimental single-case AB design was performed, using the parametric method of Allison and Gorman (1993) as a method of analysis to evaluate the effect of the intervention. This method was suggested and applied automatically by the BEHAVE application according to the number of observations made in the baseline and treatment phases. The basic assumption for carrying out this analysis has been respected (*cov* > 0). The parametric analysis was applied to evaluate the effect of a possible change in frequency levels by considering the effect of the treatment under two different aspects: the first is the potential effect of treatment on the average change in frequency of mands between the baseline and treatment phases (effect on the levels); the second is the potential effect concerns the change in trajectory that the target behavior can assume when passing from the baseline to the treatment phase (effect on the slopes). These two effects, indicating the frequency of the behavior changes in average terms and trajectory, have been estimated also considering the natural trend of the behavior of concern. The expression “natural trend” is intended to mean how the behavior of concern would have evolved naturally without any intervention. At this point, this estimate is subtracted from the observations made, obtaining a new variable called the de-trend score. This new variable expresses the frequency levels of the behavior, net of the variations that it would have naturally assumed over time. Using the de-trend score, it is possible to estimate the effect of the treatment, keeping the natural trend of our dependent variable under control.

During the treatments, both children show a progressive and linear increase in the number of correct mands over time.

**Figure 4:**
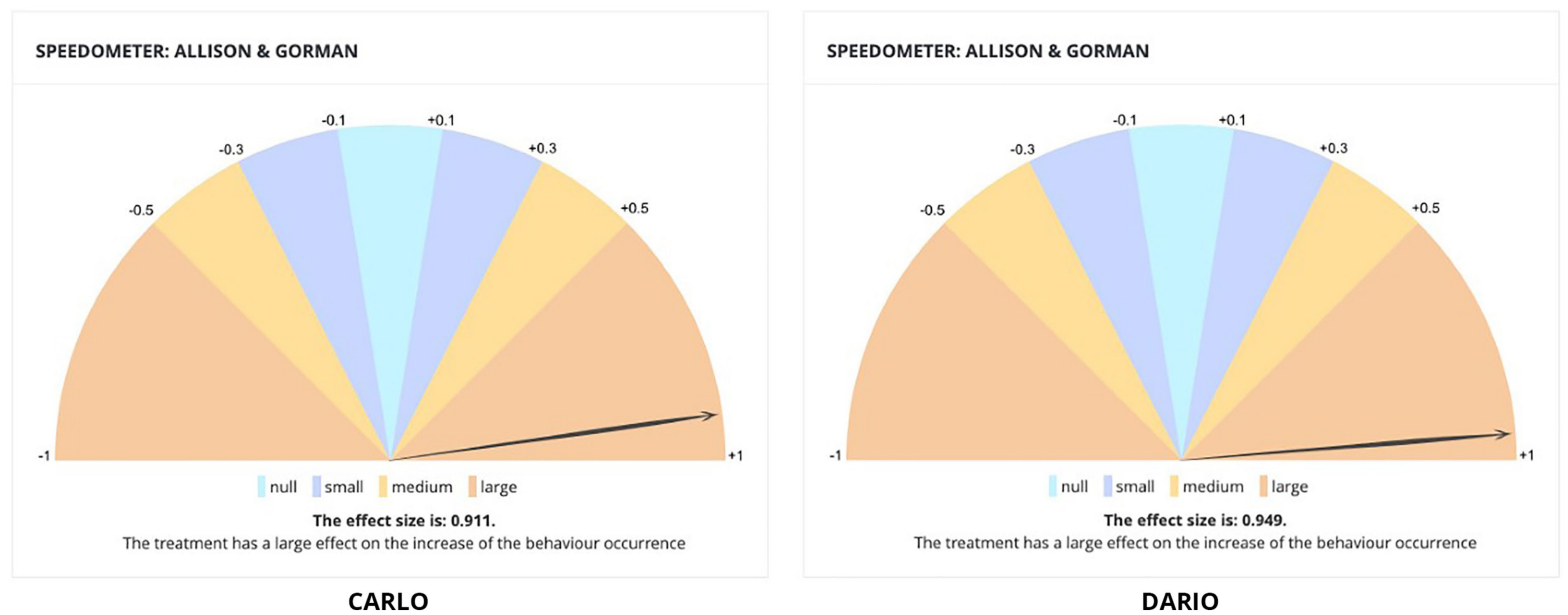
Screenshot of the BEHAVE application displaying the automatic calculation of the effect size of the interventions.

The effect size values indicate a large impact of the treatment in increasing the mand repertoire during the therapy sessions both for Dario (*r = 0.95*) and Carlo (*p = 0, r = 0.91*). The BEHAVE application summarizes the data relating to the values of the effect sizes through the speedometers shown in Figure 4. The treatment had a significant effect for both Dario (*R*^2^ = 0.94, *F*(2, 32) = 143.33, *p < 0.05*) and Carlo (*R*^2^ = 0.83, *F*(2, 32) = 77.99, *p < 0.05*).

### 5.2. Intervention versus generalization

The results obtained in the generalization phases were different for the two children. Dario has immediately started to generalize the skills learned during the intervention, showing a progressive and linear increase in the frequency of mand issued at home. Carlo, on the other hand, generalized the skills acquired in the family context after specific parent training.

During the 10 generalization sessions at home, the mand ability showed a growing trend in both children.

The analysis of the parents’ interviews showed that the children still use the repertoire of mands learned with their parents, grandparents, and in the school context. Dario show mands even when the motivating object is not in sight, while Carlo only when the items he wants are visible in his environment. Finally, the parents report an increase in communicative intentionality, reporting that children show greater interest in objects and people in the environment and begin to spontaneously manifest approximations of requests for motivating objects on sight not trained yet.

## 6. Discussion

Bringing up children with NDDs involves several issues that affect all the educational agents involved: parents and family of origin, caregivers, and teachers. Parents of children with disabilities generally have higher levels of stress than parents of typically developing children (Meadan et al., 2010; Karst and Van Hecke, 2012; Dykens, 2015). Similarly, working with children with NDDs can expose teachers, especially special needs teachers, to high levels of stress (Ghani et al., 2014) and unpleasant emotions which can eventually lead to burnout (Abel and Sewell, 1999; Richards et al., 2018). In general, teachers are stressed the most by behaviors that can hurt others, such as kicking, hitting, or biting (Amstad and Müller, 2020).

Many studies showed that ABA inclusive interventions may be effective in supporting the skills enhancement of children with ASD (e.g., Camargo et al., 2014) reducing risk factors not only for the child but for the entire family and educational network that cares for the child.

The present study explored the possibility to use the BEHAVE web application as a technological tool supporting an ABA inclusive intervention on two children with severe language impairments. Results revealed that the interventions significantly increased the mand repertoire of both children and that the technological solution facilitated the collection of reliable data not only by experienced therapists but also by caregivers in the home contest. As pointed out in the literature (Aktaş and Ciftci-Tekinarslan, 2018), this generalization seems to have been facilitated by the active participation of the carers, trained through careful parent training both to implement the procedure and to record the data with BEHAVE application, which made their collection easier. The BEHAVE application has facilitated the gathering of data in various contexts and the sharing of the results between the various figures involved.

Moreover, in the case of Carlo, the BEHAVE application allowed therapists to quickly identify the sign of difficulties in the generalization of results at home and to intervene promptly to guide his parents to maintain the skills learned in the clinical setting. These results are consistent with findings from other studies enhancing psychological interventions for subjects with NDDs through technologies (e.g., Hetzroni and Tannous, 2004). Artoni and colleagues, for example, identified positive improvements in all children participating in technological-based intensive ABA interventions, in both communication and socialization areas (Artoni et al., 2018).

The features of the BEHAVE application overcame some of the known limitations of the use of computation technologies in ABA. Many technologies do not permit monitoring the child’s activities at home and are not fully customizable, forcing users to use activities or stimuli that are already in the system (Trevisan et al., 2019; Lopez-Herrejon et al., 2020). The possibility of remote monitoring may constitute added value at a time when the COVID-19 pandemic has limited face-to-face meetings, including concerning activities for the management of neurodevelopmental disorders. Moreover, the flexibility of the measure creation of the BEHAVE application partially fulfills this need even if other features could make it more flexible in future releases of the system (e.g., the possibility to observe more than one behavior at a time). The fact that the BEHAVE application provides users with automatic statistical analyses does not imply that the application can be used without specific training or that the decisions about behavioral interventions can be completely delegated to algorithms. In this regard, the BEHAVE project produced many educational contents and was carried out both face-to-face (in five European countries) and virtual training attended by hundreds of teachers from a lifelong learning perspective. Technological tools supporting the monitoring of behaviors must foster ethics (Mittelstadt et al., 2016), transparency and integrity of data and processes, and promote awareness, knowledge, and collaboration between practitioners and caregivers to apply the best multidisciplinary treatment plan (Guinchat et al., 2020), maximizing children participation in a typical home and school setting, reducing the impact of their symptoms but also facilitating the management of the disorder by various educational agents involved in their education.

## 7. Conclusion

The results of the study showed that the BEHAVE application can be a useful tool supporting the data collection, monitoring, and analysis of behavioral interventions through SCEDs. The paper presented two cases of children with NDDs and severe language improvements that improved their mand repertoire through an ABA intervention and generalized these results also in the home and education environment.

## Data availability statement

The datasets presented in this study can be found in online repositories. The names of the repository/repositories and accession number(s) can be found at: https://figshare.com/articles/dataset/data_CSV/19753639; https://doi.org/10.6084/m9.figshare.19753639.v1.

## Ethics statement

Ethical review and approval was not required for the study on human participants in accordance with the local legislation and institutional requirements. Written informed consent to participate in this study was provided by the participants' legal guardian/next of kin.

## Author contributions

GM, AC, NS, and SP contributed to the formulation of the idea and the design of the study. GM, GC, and DT developed the software. NF, SP, and LP carried out the interventions and gathered the clinical data. NS performed the statistical analyses. GM, AC, GC, DT, LS, NS, and SP wrote the first draft of the manuscript. PD reviewed the manuscript. All authors contributed to the article and approved the submitted version.

## Conflict of interest

The authors declare that the research was conducted in the absence of any commercial or financial relationships that could be construed as a potential conflict of interest.

## Publisher’s note

All claims expressed in this article are solely those of the authors and do not necessarily represent those of their affiliated organizations, or those of the publisher, the editors and the reviewers. Any product that may be evaluated in this article, or claim that may be made by its manufacturer, is not guaranteed or endorsed by the publisher.
